# A Pilot Study of Individual Muscle Force Prediction during Elbow Flexion and Extension in the Neurorehabilitation Field

**DOI:** 10.3390/s16122018

**Published:** 2016-11-29

**Authors:** Jiateng Hou, Yingfei Sun, Lixin Sun, Bingyu Pan, Zhipei Huang, Jiankang Wu, Zhiqiang Zhang

**Affiliations:** 1 Sensor Networks and Applications Research Center (SNARC), University of Chinese Academy of Sciences (UCAS), Beijing 101408, China; houjiateng13@mails.ucas.ac.cn (J.H.); sunlixin12@mails.ucas.ac.cn (L.S.); panbingyu14@mails.ucas.ac.cn (B.P.); jkwu@ucas.ac.cn (J.W.); 2 School of Electronic and Electrical Engineering, University of Leeds, Leeds LS2 9JT, West Yorkshire, UK; Z.Zhang3@leeds.ac.uk

**Keywords:** elbow, muscle force, neuromusculoskeletal (NMS) model, neurorehabilitation, surface electromyography (sEMG)

## Abstract

This paper proposes a neuromusculoskeletal (NMS) model to predict individual muscle force during elbow flexion and extension. Four male subjects were asked to do voluntary elbow flexion and extension. An inertial sensor and surface electromyography (sEMG) sensors were attached to subject's forearm. Joint angle calculated by fusion of acceleration and angular rate using an extended Kalman filter (EKF) and muscle activations obtained from the sEMG signals were taken as the inputs of the proposed NMS model to determine individual muscle force. The result shows that our NMS model can predict individual muscle force accurately, with the ability to reflect subject-specific joint dynamics and neural control solutions. Our method incorporates sEMG and motion data, making it possible to get a deeper understanding of neurological, physiological, and anatomical characteristics of human dynamic movement. We demonstrate the potential of the proposed NMS model for evaluating the function of upper limb movements in the field of neurorehabilitation.

## 1. Introduction

Hemiplegia is a common effect of brain injury or stroke, which may have significant impact on upper limb function [1]. Patients who suffer from hemiplegia usually have difficulty performing activities of daily living (ADL) such as feeding themselves, washing themselves, dressing, etc. A proportion of patients can recover some degree of useful function in their upper limb following active rehabilitation, but the majority of them cannot retirn to their full motor capability and independence. Fortunately, neuroscience research has yielded a great deal of information on the nature of experience-dependent brain plasticity, and there is reason for optimism that our understanding of this can be capitalized upon to improve functional outcome after brain damage [2]. Therefore, quantitative evaluation of patient upper limb impairment and personalized rehabilitation treatment play important roles in the patient’s recovery process.

Thus far, three main approaches, namely assessment scales, movement evaluation, and surface electromyography (sEMG) analysis, are widely applied for objective evaluation of the upper-extremities. Assessment scales such as the Fugl-Meyer Assessment (FMA) [3], the Box & Block Test (BBT) [4], and the Action Research Arm Test (ARAT) [5], having been used for years, are all comprehensive and quantitative [6]. As these assessments are viewed and scored by therapists, the evaluation results are often subjective and inaccurate. Movement evaluation methods using motion capture systems can provide objective data on the physical movement of the upper limb or how the upper limb moves through space. These precise data may add value in monitoring progress of the patient and the rehabilitation program [6]. However, this method cannot account for neuromuscular characteristics in patients, and the neurological mechanisms used to compensate for the problems associated with their pathology are still unavailable. sEMG analysis has been investigated a lot nowadays. Recent experiments have suggested that the central nervous system (CNS) makes use of muscle synergies as a neural strategy to simplify the control of a variety of movements [7]. These synergies can compensate mechanical disabilities, resulting invisible to an external evaluation [8]. Therefore, the analysis of muscle synergies extracted from sEMG becomes an emerging technique in the field of neurorehabilitation. Although sEMG analysis is useful in the assessment of upper limb function, it is inadequate for quantitative evaluation due to lack of deep muscle's activation information and noise contamination from cross-talk and movement artifacts.

The ability to use electrophysiological recordings to inform biomechanical models enables accessing a broader range of neuromechanical variables than analyzing electrophysiological data or movement data individually [9]. Muscle force is one of these neuromechanical variables, and it opens up the possibility of examining the outcome of muscle deficiencies and to investigate causes of joint instability as encountered in clinical practice [10]. Given this, we present a neuromusculoskeletal (NMS) model to predict individual muscle force during voluntary elbow flexion and extension by fusion of sEMG and motion data. Compared with the state-of-the-art EMG-driven muscle force prediction method, our model adds a muscle excitation adjustment component to reduce the disadvantage of the effect of uncertainties in sEMG signals. Through our method, therapists can get to know patients’ specific functional muscle weakness, making it possible to conduct customized rehabilitation therapy for the patients. Thus, we demonstrate the potential of using our NMS model for evaluating the function of upper limb in the field of neurorehabilitation. In this paper, we explicate the structure of our NMS model and how it is implemented to estimate individual muscle force. We then investigate if our model can predict individual muscle force accurately with the ability to cover subject-specific joint dynamics and neural control solution.

## 2. Materials and Methods

**Figure 1:**
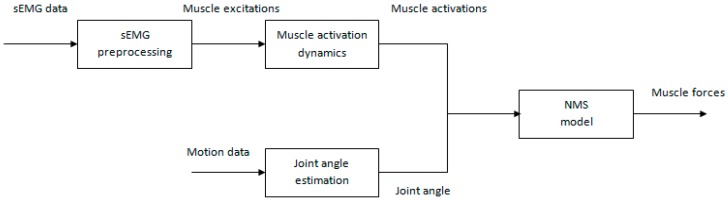
The flowchart of our muscle force prediction method. It consists of four main parts: sEMG preprocessing, muscle activation dynamics, joint angle estimation, and NMS model.

Our NMS model takes joint angle and muscle activations as inputs to estimate individual muscle force. First we describe data collection and preprocessing procedure. Second, we explicate how to get joint angle using an inertial sensor. Third, we describe how to obtain muscle activations from the preprocessed sEMG signals. Lastly, we present a detailed description about how our NMS model works to estimate muscle force. A brief flowchart of our muscle force prediction method is shown in Figure 1.

### 2.1. Data Collection and Preprocessing

Four healthy male subjects (age: 24.8 ± 1.7 years, height: 175.3 ± 4.5 cm, mass: 68 ± 7.3 kg) volunteered for this investigation and gave their informed, written consent. The project was approved by the Human Research Ethics committee at the University of Chinese Academy of Sciences. The subject sat on a chair with the upper arm kept vertical. The shoulder was orientated at 0° of flexion, 0° of abduction, and neutral rotation. The forearm was in a supinated position and the wrist was in a neutral position with the hand completely relaxed. During the test, the subject slowly flexed his forearm from a neutral position (about 10° in the sagittal plane) to fully flexed position (about 130° in the sagittal plane), and then extended back to the neutral position, while the humerus was in a neutral position and the hand remained supine. Each subject repeated the voluntary elbow flexion–extension twice to get two sets of data, one for calibration and the other for simulation.

The motion data of elbow flexion and extension was collected (50 Hz) using an MPU-9150 (InvenSense Inc., San Jose, CA, USA) sensor attached to the lateral side of the forearm. Simultaneously, sEMG signals including long head of biceps brachii, brachioradialis, and lateral head of triceps brachii were recorded (1000 Hz) by the ME6000 multi-channel bipolar sEMG recording system (Mega Electronics Ltd., Kuopio, Finland). The electrodes were placed longitudinally with respect to the muscle fiber directions and the positions of each muscle according to the recommendations from the SENIAM (surface electromyography for the non-invasive assessment of muscles) conventions [11]. A ground electrode was placed over the acromion. Traditional manual muscle test techniques [12] were adopted to obtain the maximum voluntary contraction (MVC) EMG values under isometric conditions. Experimental muscle excitations were calculated from raw EMG signals that were high-pass filtered (30 Hz), full-wave-rectified, and low-pass filtered (6 Hz) using a zero-lag fourth-order recursive Butterworth filter [13]. Experimental muscle excitations were then normalized with the peak MVC EMG values respectively.

### 2.2. Joint Angle Estimation

**Figure 2:**
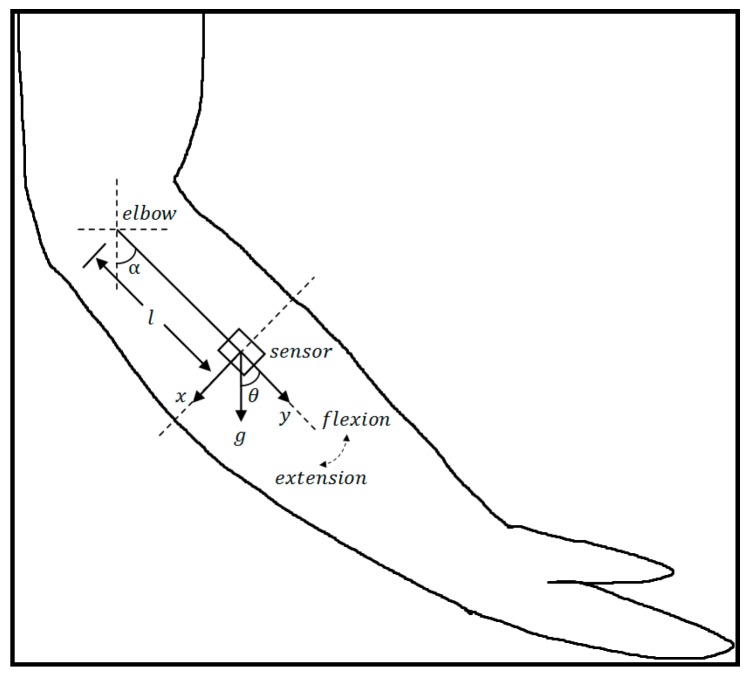
Location of the inertial sensor. The sensor was attached to the lateral side of the forearm. α is the elbow joint angle of flexion and extension. *x* and *y* represent the sensor’s X-axis and Y-axis respectively. *l* is the distance between the sensor and elbow. g represents the earth gravity. The elbow join angle can be obtained by fusing the sensor’s acceleration and angular rate.

The joint angle can be obtained by the integration of the angular rate using a gyroscope. However, as the result of integration, the errors also accumulate. This results in a drift over a period of time [14]. To overcome this problem, we employed a Kalman filter which fused angular rate and acceleration to obtain joint angle since Kalman filter provides a convenient, efficient, and elegant framework for combining different types of measurements to recover the state of a given system [15]. Location of the inertial sensor is shown in Figure 2. The sensor was attached to the lateral side of the forearm. α is the elbow joint angle of flexion and extension, which is equal to θ, the angle between sensor’s Y-axis and the direction of gravity. Thus, in the following part, we take θ as the joint angle.

We define the state vector x by using three types of parameter, namely, the joint angle θ, the joint angular rate $\dot{\theta }$, and the joint angular acceleration $\ddot{\theta }$. The process model is used to predict the evolution of the state at time step k from time step k−1, so the state vector is given as $x_{k}={\left[\begin{array}{ccc} \theta_{k} & {\dot{\theta }}_{k} & {\ddot{\theta }}_{k} \end{array}\right]}^{T}$. In line with the Continuous Wiener Process Acceleration (CWPA) model [16], the angular acceleration of elbow is assumed changing little within a short sampling period τ. Therefore, the process model can be represented by the linear difference equation:

(1) $$x_{k}=Ax_{k-1}+n_{k-1},$$

where $A=\left[\begin{array}{ccc} 1 & \tau & \frac{\tau^{2}}{2}\\ 0 & 1 & \tau \\ 0 & 0 & 1 \end{array}\right]$, $n_{k}$ is the Gaussian white process noise with zero mean and covariance matrix Q.

The measurement model relates the measurement vector z to the state vector x. The wearable inertial sensor provides two types of measurement: angular rate and acceleration. The generalized form of the measurement equation is given as

(2) $$z_{k}={\left[\begin{array}{ccc} a_{x,k} & a_{y,k} & \omega_{x,k} \end{array}\right]}^{T}=h\left(x_{k},m_{k}\right),$$

where $a_{x,k}$ is the accelerometer reading of X-axis at time step *k*, $a_{y,k}$ is the accelerometer reading of Y-axis, $\omega_{x,k}$ is the gyroscope reading of X-axis, $m_{k}$ represents the Gaussian white measurement noise with zero mean and covariance matrix R. We can rewrite the measurement equation to make it more clear:

(3) $$a_{x,k}=-gsin\theta_{k}+{\ddot{\theta }}_{k}l+m_{1,k}\;a_{y,k}=-gcos\theta_{k}+{{\dot{\theta }}_{k}}^{2}l+m_{2,k},\;\omega_{x,k}={\dot{\theta }}_{k}+m_{3,k},$$

where g is the magnitude of earth gravity, l is the distance between elbow and sensor. ${\ddot{\theta }}_{k}l$ and ${{\dot{\theta }}_{k}}^{2}l$ are the tangential and normal acceleration respectively.

As the measurement relationship to the process is nonlinear, we adopt an extended Kalman filter (EKF) to deal with the nonlinearity. The time update equations are given as follows:

(4) $${\hat{x}}_{k}^{-}=A{\hat{x}}_{k-1}\;P_{k}^{-}=AP_{k-1}A^{T}+Q,$$

The measurement update equations are described as follows:

(5) $$K_{k}=P_{k}^{-}H_{k}^{T}{\left(H_{k}P_{k}^{-}H_{k}^{T}+R\right)}^{-1}\;{\hat{x}}_{k}={\hat{x}}_{k}^{-}+K_{k}\left(z_{k}-h\left({\hat{x}}_{k}^{-},0\right)\right),\;P_{k}=\left(I-K_{k}H_{k}\right)P_{k}^{-}$$

where $H_{k}$ is the partial derivative matrix

(6) $$H_{k}={\frac{\partial h}{\partial x_{k}}|}_{{\hat{x}}_{k}^{-}}=\left[\begin{array}{ccc} -gcos{\hat{\theta }}_{k}^{-} & 0 & l\\ gsin{\hat{\theta }}_{k}^{-} & 2{\hat{\dot{\theta }}}_{k}^{-}l & 0\\ 0 & 1 & 0 \end{array}\right].$$

The update process is repeated at each time step and the values of the state vector are estimated. Thus, we can get elbow joint angle during flexion and extension.

### 2.3. Muscle Activation Dynamics

EMG is a measure of the electrical activity that is spreading across the muscle, causing it to activate. This results in the production of muscle force. However, it takes time for the force to be generated—it does not happen instantaneously [17]. To this end, we adopted a second-order discrete linear model [18] to model neural activation from muscle excitation obtained through preprocessing, in the form of a recursive filter:

(7) $$u\left(t\right)=\alpha e\left(t-d\right)-\left(C_{1}+C_{2}\right)u\left(t-1\right)-C_{1}C_{2}u\left(t-2\right),$$

where e(t) is the muscle excitation at time *t*, u(t) is the neural activation, α is the muscle gain, $C_{1}$ and $C_{2}$ are recursive coefficients, and d is the electromechanically delay.

The neural activations were then adjusted to account for either a linear or non-linear EMG-force relationship [18]:

(8) $$a\left(t\right)=\frac{e^{Au\left(t\right)}-1}{e^{A}-1},$$

where a(t) is the muscle activation, u(t) is the neural activation, and A is the non-linear shape factor.

Initial values of these system parameters were set according to the previous work [19]. A calibration process was then conducted to make our model match the subject-specific physiological characteristics. More details about calibration are given in the Section 2.4.

In our model, we selected short head of biceps (BICshort), long head of biceps (BIClong), brachialis (BRA), brachioradialis (BRD) as four elbow flexors and long head of triceps (TRIlong), medial head of triceps (TRImed), lateral head of triceps (TRIlat) as three elbow extensors. Selection of these muscles was based on computing and comparing the potential moment contribution among the 24 muscles wrapping across the elbow joint [20]. As we only collected sEMG signals of BIClong, BRD and TRIlat, we adopted a neural mapping method [13] to get the other four muscles' activations. To be specific, we assumed that BICshort has the same activation as BIClong and three heads of triceps have the same activation. Considering that the EMG signal of BRA cannot be measured by the non-invasive methods, we assumed that it has the same activation of BIClong. These assumptions were testified feasible in previous study [19].

### 2.4. NMS Model

The NMS model is the core of our muscle force prediction method. It consists of four main components: muscle kinematics, muscle contraction dynamics, joint moment estimation, and static optimization.

**Figure 3:**
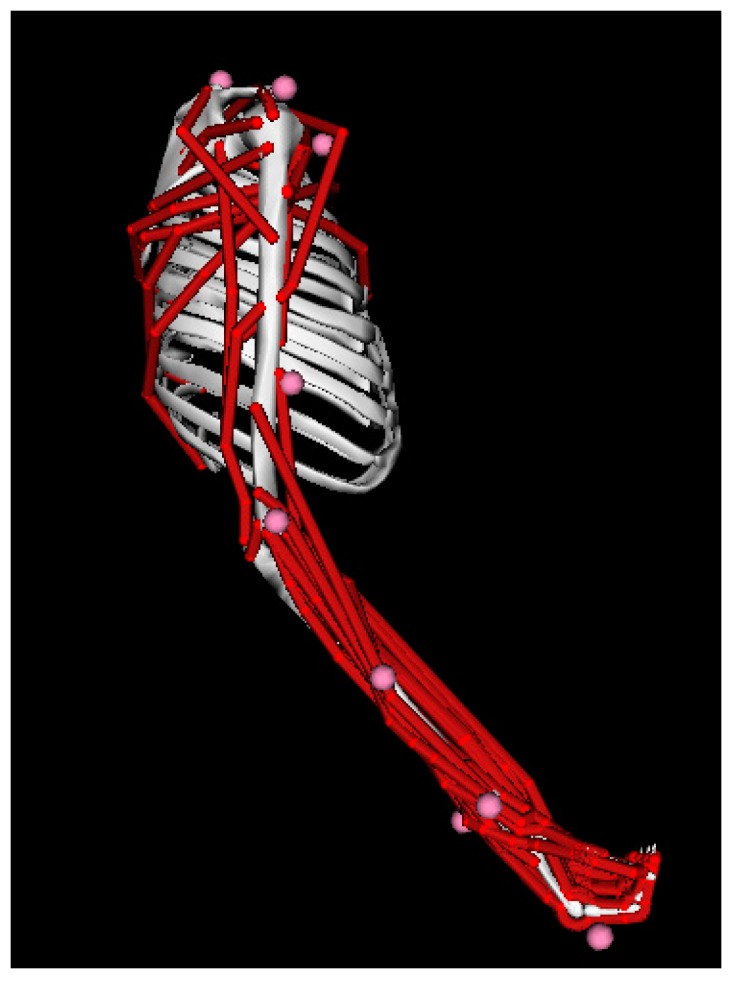
OpenSim upper limb musculoskeletal model. This model was developed by Saul et al. [19]. It consists of 7 body segments and 32 muscles across the shoulder, elbow, forearm, and wrist. The muscle kinematics parameters and experimental joint moment can be obtained through this musculoskeletal model.

Muscle kinematics transforms the joint angle into muscle kinematics parameters such as musculotendon unit (MTU) lengths and moment arms using an upper limb musculoskeletal model [19] based on OpenSim [21]. This upper limb musculoskeletal model is shown in Figure 3, which should be scaled to match the subject’s anthropometry first. We also employed the OpenSim Inverse Dynamics Tool to get the experimental joint moment. More details about scale and Inverse Dynamics Tool are described in other articles [21,22]. In general, OpenSim upper limb model characterizes the transformation from joint angle to muscle kinematics parameters, which is a necessary prerequisite to individual muscle force prediction.

Muscle contraction dynamics transforms the muscle activation and muscle kinematics parameters into muscle force:

(9) $$F^{m}=F^{max}\left[f_{a}\left({\tilde{l}}_{m}\right)f_{v}\left({\tilde{v}}_{m}\right)\right]a+f_{p}\left({\tilde{l}}_{m}\right)+d_{m}{\tilde{v}}_{m}]cos\varphi ,$$

where $F^{m}$ is the muscle force, $F^{max}$ is the maximum isometric muscle force, ${\tilde{l}}_{m}$ is the normalized muscle fiber length, ${\tilde{v}}_{m}$ is the normalized muscle fiber contraction velocity, a is the muscle activation, $d_{m}$ is the muscle damping element, φ is the pennation angle. $f_{a}\left({\tilde{l}}_{m}\right)$ is the active force-length relation that expresses the ability of muscle fibers to produce force at different lengths, $f_{p}\left({\tilde{l}}_{m}\right)$ is the passive force-length relation that represents the force response of the fibers to strain, $f_{v}\left({\tilde{v}}_{m}\right)$ accounts for the force contribution of the fiber contraction velocity.

In joint moment estimation, the force generated by each muscle is multiplied by the respective moment arm and then summed to yield the estimated joint moment:

(10) $$\hat{\tau }=\sum_{i=1}^{N_{muscles}}\left(MA_{i}\times {F^{m}}_{i}\right),$$

where $\hat{\tau }$ is estimated joint moment, $N_{muscles}$ is the number of muscles, $MA_{i}$ is the moment arm of muscle i, ${F^{m}}_{i}$ is the force of muscle i.

**Figure 4:**
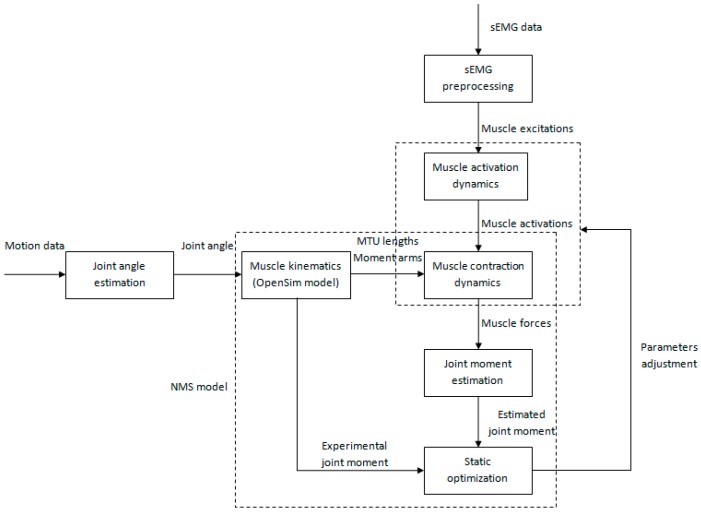
The flowchart of calibration process. Static optimization is used to adjust system parameters in muscle activation dynamics and muscle contraction dynamics.

Static optimization was used to adjust system parameters in muscle activation dynamics and muscle contraction dynamics. This calibration process aimed at matching the subject’s specific EMG-force generating properties. A simulated annealing algorithm [23] was used to minimize the root mean square error (*RMSE*) between the experimental and estimated joint moment by varying parameters within pre-defined boundaries. The entire flowchart of calibration is shown in Figure 4.

**Figure 5:**
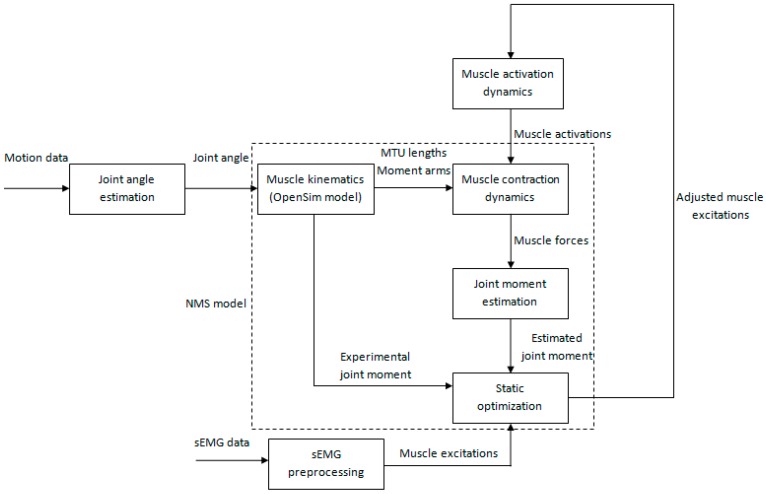
The flowchart of simulation process. Static optimization is used to adjust muscle excitations to make the joint moment estimation more close to reality.

Following calibration, we employed a novel set of trail as input to drive the calibrated model. This simulation process is shown in Figure 5. Unlike calibration, the simulation process took adjusted muscle excitations as inputs of the muscle activation dynamics. This adjustment mechanism aimed at reducing the disadvantage of the effect of uncertainties in sEMG signals. Adjusted muscle excitations can be obtained using the following static optimization method:

(11) $$min\;F_{obj}=\left|\tau -\hat{\tau }\right|\;s.t.\;\left|\frac{e_{i}-{\hat{e}}_{i}}{e_{i}}\right|<Th\;\;\forall i\in N_{muscles},$$

where τ is the experimental joint moment, $\hat{\tau }$ is the estimated joint moment, $e_{i}$ is the experimental muscle excitation for muscle i, ${\hat{e}}_{i}$ is the adjusted muscle excitation for muscle i, Th is the threshold constrained in the interval (0, 1), and $N_{muscles}$ is the number of muscles. Considering the noise contamination from cross-talk and movement artifacts is very difficult to be removed in EMG signal, and true EMG-maxima used in normalization is difficult to attain, excessive pursuit of muscle excitation tracking accuracy may limit the model’s ability in predicting individual muscle force and joint moment. Therefore, our method allows a tolerance in muscle excitation tracking accuracy to make the joint dynamics closer to reality. Th is the only parameter we need to tune in the static optimization. Larger Th value means muscle excitation can be adjusted in a wider range, which may improve the accuracy of joint moment estimation. How to determine Th value is discussed in the rest of this section.

First, two parameters, namely, the coefficient of determination ($R^{2}$) and the normalized root mean squared deviation (*NRMSD*) [24], are introduced to measure the similarity between the estimated and experimental results. The form of *NRMSD* is given as:

(12) $$NRMSD=\frac{\sqrt{\frac{1}{N}\sum_{i=1}^{N}{\left({\hat{X}}_{i}-X_{i}\right)}^{2}}}{max\left(\hat{X},X\right)-min\left(\hat{X},X\right)},$$

where X and $\hat{X}$ refer to the two variables being compared, N refers to the number of points in the data set.

According to the previous study [24], comparison results with 0.0 ≤ NRMSD ≤ 0.3 and $0.7\;\leq R^{2}\leq \;1.0$ are thought to be acceptable for joint moment estimation. In our approach, we narrowed them down to 0.0 ≤ NRMSD ≤ 0.04 and $0.95\;\leq R^{2}\leq \;1.0$ to make the NMS model more elaborate. We first set Th = 0.1, then increased it with a 0.1 step size until the joint estimation result achieved the proposed criterion. Validation of this adjustment method is discussed in the Results section. 

### 2.5. Validation Procedures

The first test assessed the accuracy of our joint angle estimation. The similarity between experimental and estimated joint angle was quantified using $R^{2}$ and *NRMSD*. Here, experimental joint angle was obtained by the MMocap system (MicroSens Ltd., Wuxi, China).

The second test assessed the feasibility of our muscle excitation adjustment method. We investigated whether a proper selection of Th value can improve the prediction of individual muscle force accounting for the realistic joint dynamics and neural control strategy. To this end, we evaluated the effects of different Th values on joint moment prediction and muscle excitation pattern tracking. *NRMSD* and $R^{2}$ were used. In addition, we adopted a non-negative matrix factorization (NMF) algorithm [25] to extract muscle synergies and then used scalar product [26] to measure similarity between the experimental synergies and the adjusted synergies. This muscle synergy analysis allows us to investigate whether our adjustment is feasible in terms of neural control strategy.

The third test compared our model with the EMG-driven model to figure out the advantage of muscle excitation adjustment mechanism. Here, EMG-driven method is a forward dynamic NMS modeling to predict muscle forces and joint moments from neural signals [17], which has been widely used these years [18,27,28]. The structure of EMG-driven model is almost the same as ours except the lack of a muscle excitation adjustment mechanism.

## 3. Results

**Figure 6:**
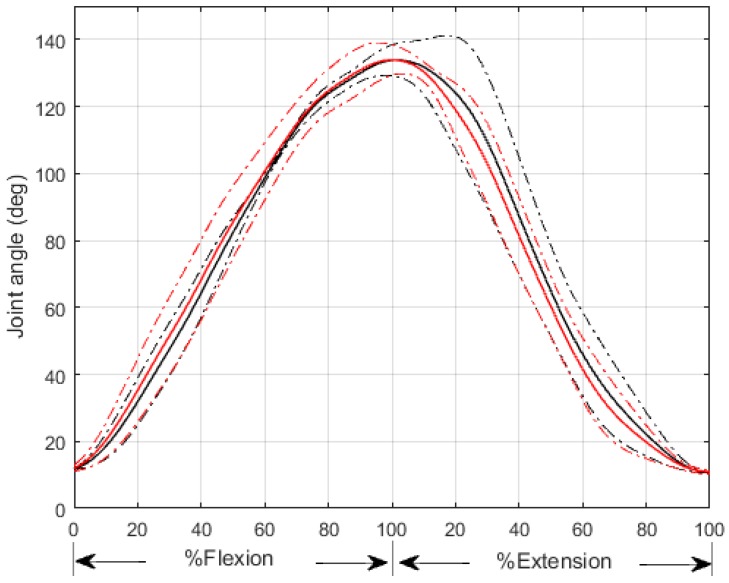
Comparison between experimental and estimated joint angle. The black and red solid lines represent the average experimental and estimated joint angles respectively. The black and red dash dotted lines represent the standard deviations of the experimental and estimated joint angle respectively. The average elbow flexion-extension duration is about 5.8 s.

The first test showed that our EKF method can predict joint angle accurately with low *NRMSD* (*NRMSD =* 0.0434 ± 0.0056) and high $R^{2}$ ($R^{2}$ = 0.9951 ± 0.0035) (see Figure 6) across four subjects. As joint angle information plays an important role in muscle force prediction, our EKF model provides an easy way to track human movement compared with a visual tracking system.

**Figure 7:**
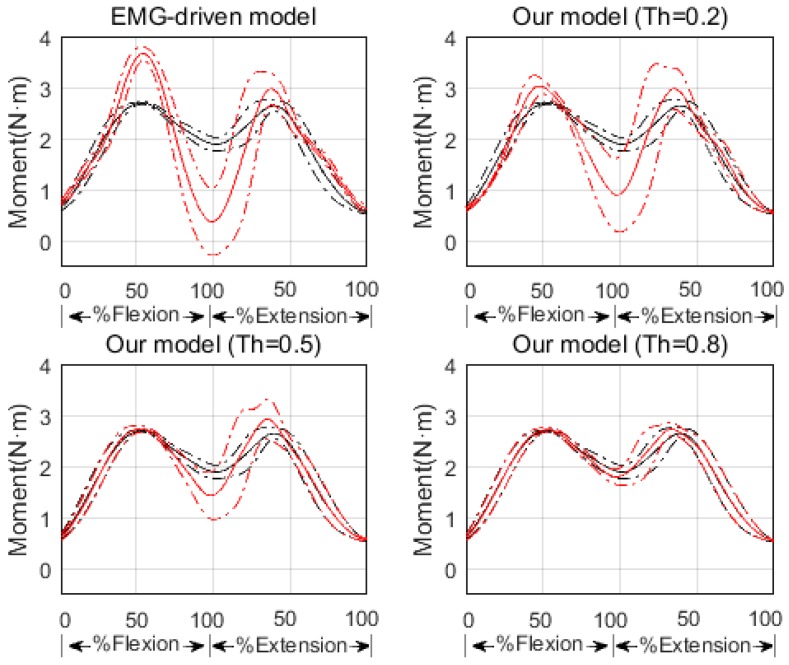
Joint moment estimation of EMG-driven model and our model with different Th values (Th = 0.2, 0.5, 0.8). The black and red solid lines represent the average experimental and estimated joint moments, respectively. The black and red dash dotted lines represent the standard deviations of the experimental and estimated joint moment, respectively.

**Figure 8:**
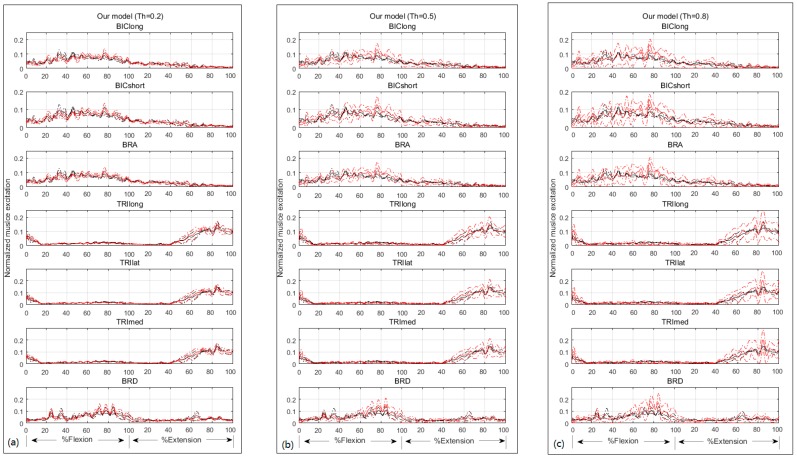
Experimental muscle excitations and adjusted muscle excitations with different Th values: (**a**) Th = 0.2; (**b**) Th = 0.5; (**c**) Th = 0.8. The black and red solid lines represent the average experimental and adjusted muscle excitations, respectively. The black and red dash dotted lines represent the standard deviations of the experimental and adjusted muscle excitations respectively.

**Figure 9:**
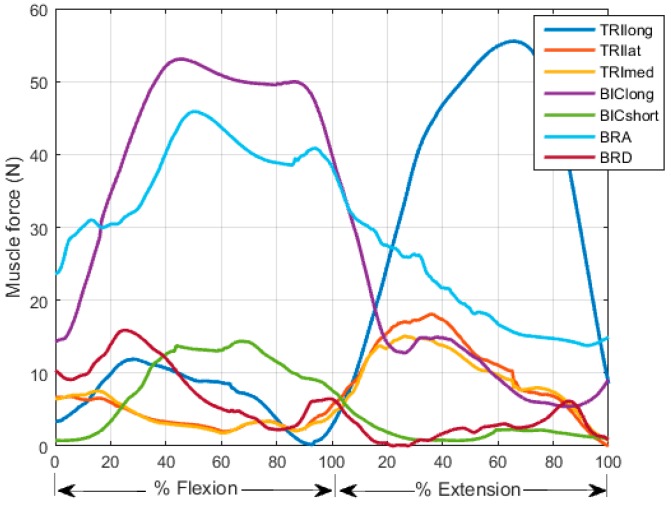
Estimation of individual muscle forces during elbow flexion and extension of one subject (Th = 0.8). BIClong and BRA are the main action muscles in the flexion phase. TRIlong is the main action muscle in the extension phase.

**Figure 10:**
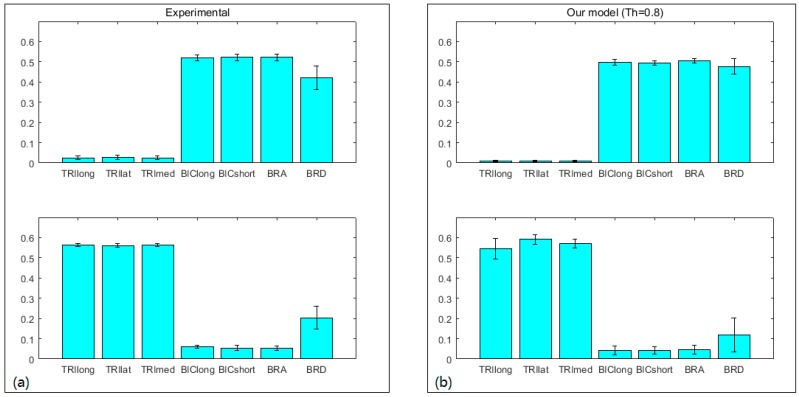
Muscle synergy analysis. NMF was used to extract muscle synergy patterns. (**a**) The experimental muscle synergy patterns; (**b**) the adjusted muscle synergy patterns (Th = 0.8). The scalar products between experimental and adjusted muscle synergy patterns are above 95% of all the four subjects.

The second test revealed that the larger Th value is, the better joint moment estimation will be (see Figure 7 and Table 1). Although muscle excitation tracking ability degrades as the Th value raises (see Figure 8 and Table 2), this degradation mainly lies in amplitude, the shape matches fairly well. Here we took one subject for example to analyze individual muscle force. According to the proposed criterion, we chose Th = 0.8. His individual muscle forces during elbow flexion and extension are shown in Figure 9. In the flexion phase, BIClong and BRA are the main action muscles, with maximum muscle force 53.07 N and 45.88 N respectively. The curves of these two muscles are both inverse U-shapes. In the extension phase, TRIlong is the main extensor, also with an inverse U-shape force curve. The other three subjects' muscle force curves are basically in line with the example one. These results of elbow dynamics show great agreement with previous work [10,29]. Through muscle synergy analysis (see Figure 10), we figure out that the scalar products between experimental and adjusted muscle synergy are all above 95% of the four subjects, meaning our method can capture the neural control strategy in spite of some accuracy loss of muscle excitation tracking. In general, we can demonstrate with a proper selection of Th value that our NMS model can predict individual muscle force accurately with the ability to cover subject-specific joint dynamics and neural control solution.

The third test showed that our NMS model improved the joint moment estimation compared with the EMG-driven model (see Figure 7 and Table 1). From the result we can see even a slight adjustment in muscle excitation can make the joint moment estimation more accurate. That is to say, our model is a feasible trade-off method to analyze joint dynamics with respect to the uncertainties in EMG signals.

## 4. Discussion

Upper limb disorder is one of the primary symptoms of stroke or other brain damage and seriously affects the patient's daily life and work. The concept and methods of rehabilitation medicine have played important roles in the patient recovery process. However, as the function of an upper limb is much more complex than a lower limb, no protocol proposed for the objective evaluation of the upper-extremities has achieved consensus [30]. Elbow motion is fundamental for positioning the hand in space since it determines how far the hand can reach [30]. It has been estimated that a 50% reduction in elbow motion could reduce upper extremity function by almost 80% [31]. Given those, we developed an NMS model to analyze individual muscle force during elbow flexion and extension. Compared with the three major approaches of upper limb evaluation that previously mentioned, our method can provide the information about how the individual muscle force changes during the movement, which is of great importance for therapists to conduct personalized rehabilitation therapy for the patients. More specifically, our model is a fusion of movement evaluation and sEMG analysis, which maintains the basic functions of both methods, while making it possible for us to get a deeper understanding of human dynamic movement mechanism.

A motion monitoring system developed for clinical use should be transportable, easy to set up, and have minimal impact on patients' normal range of movement [6]. To date, optical motion capture is one of the most mature solutions among all existing motion capture methods [32] in the fields of animation production, digital filmmaking, and sports training, etc. However, setting up optical systems is very complex and time consuming, which limits its application in rehabilitation medicine since patients usually have difficulties in performing daily activities. In our method, we collected the acceleration and angular rate of an inertial sensor which was attached to the lateral side of the forearm. As the inertial sensor is small and light, our method will not cause discomfort for the patients during the experiment. From the result of the first test, we can see our EKF method can predict elbow flexion and extension angle precisely with low *NRMSD* and high $R^{2}$. We demonstrate the potential of using our EKF model to calculate joint angle during shoulder abduction or flexion. However, our method of joint angle estimation applies to one degree of freedom (DOF) only, which is a limitation that should not be neglected. For multi-DOF movement evaluation, a more elaborate joint angle estimation model should be developed.

The purpose of muscle activation dynamics is to determine muscle activations from muscle excitations, accounting for the way EMG is related to force. Muscle excitation is the normalized, rectified, and filtered EMG values, ranging from zero (no activity) to one (full activity). This preprocessing of raw EMG signals aims at removing noise caused by skin or electrode movement and allowing comparisons between muscles in spite of the differences in types of electrodes used, the gain of the amplifiers, etc. Considering the muscle twitch response and linearity or non-linearity EMG–force relationship, we used a second-order discrete linear model and an exponential function to obtain muscle activations. Muscle activation dynamics is proven to be a mature method in current movement neuroscience and biomechanics research [13,17,18,27,33]. In our approach, we used a neural mapping method [13] to generate muscle activations for all seven muscles. This is based on the hypothesis that two muscle groups which shared the same innervation and contributed to the same mechanical action were assumed to have the same EMG pattern [34]. This assumption was found to be feasible in a previous elbow flexion-extension experiment [19]. In our method, muscle excitations are adjusted in a pre-defined range, resulting in joint moment closer to the reality. This adjustment may boost the adoption and development of the mapping technique. Figure 10 shows the experimental and adjusted muscle synergy patterns, which are in great agreement with the previous study [35]. However, their study did not cover muscle BRA. Compared with the data cited from Koo and Mak [36] which was measured using both surface and fine electrodes, the pattern of BRA excitation as well as other muscles of our experiment matched well with theirs. In this way we tested the validity of the muscle mapping method in the elbow flexion-extension experiment.

In our NMS model, a Hill-type musculotendon model was used to calculate individual muscle force. This Hill-type model is most widely used in muscle-driven simulations of human and animal movements because of its computational simplicity and close relation to commonly measured experimental variables [37]. From the result of the third test, we can see the accuracy of joint moment estimation is poor (*NRMSD* = 0.1847 ± 0.0199 and $R^{2}$ = 0.5653 ± 0.1225) when taking unadjusted experimental muscle excitations as inputs. This is mainly because noise contamination in the EMG signal from cross-talk and movement artifacts is very difficult to remove and neural mapping is only a simplification method to compensate for the lack of deep muscle's excitation. Moreover, obtaining true EMG maxima from maximal voluntary efforts is a critical step and a source of uncertainties [38]. Given those, we developed a hybrid NMS model that incorporated EMG-driven model and static optimization. In this, the joint moment tracking error is minimized by adjusting muscle excitation within a reasonable boundary that matches the temporal EMG pattern using a static optimization method. Through the second test, we can see the precision of joint moment estimation improves as the Th value raises. Although the muscle excitation tracking ability degrades as the Th value becomes closer to 1, the shape of the adjusted muscle excitation still shows good agreement with the experimental muscle excitation. What's more, from the muscle synergy analysis perspective, the scalar products between the experimental and adjusted muscle synergy are above 95% for all four subjects, meaning these adjusted muscle excitations still can reflect the realistic neural control strategy. Thus we can demonstrate with a proper selection of Th value that our model can predict individual muscle force accurately with the ability to reflect subject-specific joint dynamics and neural control solutions. Thanks to the calibration and muscle excitation adjustment mechanism, our NMS model has the ability to be applied to other joints such as shoulder, knee, ankle, etc. Taking the knee for example, the main process is basically the same as the elbow’s, while two things should be noted. First, the purpose must be specified, whether it’s for gait analysis with ground contact or just knee flexion extension experiment without ground contact. If the purpose is the former one, an in-ground force plate should be brought in to measure ground reaction force, which is an input of OpenSim inverse dynamic tool to calculate the experimental joint moment. Second, the choice of muscles should be investigated carefully. As there may be more muscles wrapping across the knee, I would suggest first figuring out each muscle’s mechanical function, and then choosing the main action muscles as the flexors and extensors in the model. As the deep muscle’s EMG cannot be measured non-invasively, I suggest taking the superficial muscle’s activation, which shares the same innervation and contributes to the same mechanical action with deep muscle as the deep muscle’s activation.

Several limitations of our study should be noted. First, as previously noted, our joint angle estimation method only applies to one DOF. In the elbow flexion-extension experiment, the inertial sensor's X-axis and Y-axis should be kept in the sagittal plane. This limits the ability of our NMS model to predict individual muscle force during multi-DOF movement. Therefore, developing a more elaborate joint angle estimation method should be the subject of our future work. Preliminarily we plan to build a three-node sensor network, with the three inertial sensors attached to the forearm, the upper arm, and the trunk. According to the previous study [39], sensors’ acceleration data can be processed to obtain both shoulder and elbow joint angles based on the hierarchical structure of the upper limb. Second, neural mapping is a simplification of muscle recruitment strategies and may result in suboptimal model calibration and inaccurate force prediction [13]. The aim of this mapping is to solve the problem that a deep muscle’s EMG cannot be measured non-invasively. Although we obtained relatively good joint dynamics estimation results using a neural mapping method, how to simulate a deep muscle's excitation requires further research. Last, our work is a pilot study with the purpose of introducing a new quantitative assessment of upper limb function based on individual muscle force into neurorehabilitation. As we only collected four subjects’ motion and sEMG data for one movement, a generalizable evaluation criterion cannot be established. Nevertheless, the scaling and calibration process make our NMS model can be applied across individuals and tasks. This allows us to perform more experiments in the future.

## 5. Conclusions

In conclusion, this work proposed an NMS model to predict individual muscle force during elbow flexion and extension, accounting for the subject-specific joint dynamics and neural control solution. Our method combines the merits of movement evaluation and sEMG analysis and shows great potential in the field of upper limb function assessment and personalized neurorehabilitation. We believe that the reliable determination of muscle forces as a function of neural drive will provide us with a deeper understanding of neurological, physiological, and anatomical characteristics of human dynamic movement.

## Figures and Tables

**Table 1:** Comparison between experimental and estimated joint moment using the EMG-driven model and our model with different Th values (the data are given as mean ± SD).

	EMG-Driven Model	Our Model (Th = 0.2)	Our Model (Th = 0.5)	Our Model (Th = 0.8)
Moment_NRMSD	0.1847 ± 0.0199	0.1217 ± 0.0419	0.0662 ± 0.0250	0.0317 ± 0.0111
Moment_$R^{2}$	0.5653 ± 0.1225	0.7544 ± 0.1729	0.8942 ± 0.1160	0.9758 ± 0.0215

**Table 2:** Comparison between experimental and adjusted muscle excitations with different Th values (the data are given as mean ± SD).

	Our Model (Th = 0.2)	Our Model (Th = 0.5)	Our Model (Th = 0.8)
Excitation_BIClong_NRMSD	0.0706 ± 0.0038	0.1202 ± 0.0134	0.1356 ± 0.0184
Excitation_BICshort_NRMSD	0.0674 ± 0.0021	0.1140 ± 0.0139	0.1352 ± 0.0132
Excitation_BRA_NRMSD	0.0709 ± 0.0035	0.1192 ± 0.0126	0.1355 ± 0.0160
Excitation_TRIlong_NRMSD	0.0436 ± 0.0100	0.0681 ± 0.0108	0.0786 ± 0.0096
Excitation_TRIlat_NRMSD	0.0412 ± 0.0088	0.0656 ± 0.0073	0.0724 ± 0.0067
Excitation_TRImed_NRMSD	0.0424 ± 0.0095	0.0684 ± 0.0091	0.0723 ± 0.0090
Excitation_BRD_NRMSD	0.0553 ± 0.0026	0.0923 ± 0.0074	0.1125 ± 0.0073
Excitation_BIClong_$R^{2}$	0.8894 ± 0.0097	0.6948 ± 0.0352	0.5906 ± 0.0945
Excitation_BICshort_$R^{2}$	0.8967 ± 0.0056	0.7381 ± 0.0342	0.6161 ± 0.0728
Excitation_BRA_$R^{2}$	0.8872 ± 0.0084	0.7161 ± 0.0389	0.5860 ± 0.0633
Excitation_TRIlong_$R^{2}$	0.9598 ± 0.0206	0.8956 ± 0.0212	0.8215 ± 0.0412
Excitation_TRIlat_$R^{2}$	0.9637 ± 0.0178	0.9037 ± 0.0198	0.8340 ± 0.0640
Excitation_TRImed_$R^{2}$	0.9614 ± 0.0201	0.8955 ± 0.0206	0.8172 ± 0.0400
Excitation_BRD_$R^{2}$	0.9139 ± 0.0221	0.7298 ± 0.0341	0.5899 ± 0.0893
